# Does Wheat Production Through Irrigation Improve Smallholder Farmers′ Food Security? Evidence From Seka Chekorsa District, Ethiopia

**DOI:** 10.1155/tswj/6692347

**Published:** 2026-05-06

**Authors:** Zanaba Adamu Asefa, Fikadu Mitiku, Yadeta Bekele Bekere

**Affiliations:** ^1^ Jimma University College of Agriculture and Veterinary Medicine Department of Agricultural Economics and Agribusiness Management, Jimma, Ethiopia; ^2^ Department of Agricultural Economics, Arsi University, Asella, Ethiopia, arsiun.edu.et

**Keywords:** Ethiopia, food security, logit, small-scale irrigation, technology adoption, wheat

## Abstract

The Seka Chekorsa District in Jimma Zone holds significant potential for irrigation, particularly for small‐scale wheat production under irrigation farming system. Recently, the Ethiopian government has given irrigation facility construction a lot in order to boost Agricultural productivity and output. However, limited research has been conducted in the adoption of small‐scale irrigation for wheat production and its impacts on household food security in the Seka Chekorsa District. The aim of this study was to examine the adoption of small‐scale irrigation wheat production and its impacts on household food security in the Jimma Zone, Seka Chekorsa district. A cross‐sectional study design was employed, integrating quantitative and qualitative data collected through survey, key informant interviews, and focus group discussions with 100 adopters and 153 nonadopters from February to April 2023. Secondary data were obtained from published and unpublished sources. The data were analyzed using descriptive and inferential statistics, Logit models, and propensity score matching. A binary logit model analysis result showed education level, family size, credit use, frequency of extension contacts, and ownership of livestock significantly and positively influenced the adoption of irrigated wheat production. However, the distance of land plot from the water source negatively affected small‐scale irrigation wheat production adoption. The propensity score matching results indicated that the adoption of irrigated wheat production raised the caloric intake of households by 518.86 kcal/AE/day compared with nonadopters. The qualitative findings indicated key constraints to small‐scale irrigation wheat production, including lack of irrigation equipment, limited access to improved seeds, high input costs, water logging, bird attacks, and untimely rainfall. But, water and labor availability were noted as opportunities. The study also found that adopters of irrigated wheat production are more food secure than nonadopters. Therefore, raising awareness among nonadopters and promoting further research in this area is recommended.

## 1. Introduction

Agricultural technology is essential for boosting farm productivity, reducing poverty, improving national food security [1, 2]. One of the key areas for intervention to enhance agricultural productivity and minimize food insecurity in the country′s rural areas is the development of irrigation. It helps smallholder farmers cope with rainfall by ensuring a reliable, sustainable water supply for both their crops and livestock [3].

Ethiopia has substantial potential and favorable agro ecological conditions for producing sufficient amounts of wheat grain for domestic consumption and export [4]. Ethiopia is the second largest wheat producer after South Africa, which is solely under rain‐fed conditions and it has huge potential for irrigation farming systems [5]. In Ethiopia, in 2022, a recorded 8.2 million tons of wheat were harvested from the nation′s 2.6 million hectares using both rained and irrigation farming systems [6].

Small‐scale irrigation wheat production technologies are among the newly familiarized crop technologies under the irrigation farming system [7]. Ethiopian government has therefore embarked on irrigation wheat production for the last 4 years. This initiative is aimed at boosting the country′s agricultural output and reduce its dependency on wheat imports. To achieve this goal, the Ethiopian government has supported and promoted irrigated wheat production in lowland and midland areas in addition to wheat production during the main rainy season.

Wheat is one of a curtail stable food and the primary food source for over 2.5 billion people worldwide [8]. Compared with other cereal crops, it provides more calories and protein to the global diet [7]. Wheat is a strategic crop for food security, import substitution, and supply of raw materials for the agro‐processing industry. In Ethiopia, it is used to make bread, biscuits, cakes, and sandwiches, among other human delicacies. Besides, wheat straw is commonly used as a roof thatching material and as a feed for animals [9]. Due to the increasing demand for food resources and the essential of producing the crop on a large scale with the use of irrigation to meet the demand, wheat is an economically significant crop in Ethiopia [10]. Using all scientifically recommended production technology has highly contributed to improving wheat production and productivity [11].

On the basis of this attention, agricultural technologies for small‐scale irrigation have been developed, and attempts have been made to hasten their adoption and spread. Therefore, the small scale irrigation wheat production is becoming increasingly imposing for expanding domestic wheat production in the lowland and highland area of the country for the farmers to stabilize yields by reducing dependence on unpredictable rainfall, mitigating the adverse effect of heat stress and optimizing water use efficiently on limited land [12, 13].

Oromia is among the country′s largest regional states in terms of arable land and irrigated wheat production [14]. Considering its land and resources, the government has given high priority to irrigated wheat production in both lowland and midland areas of the region. In Jimma, the Seka Chekorsa district has high irrigation possible in terms of extensive land area and favorable land for irrigation. The Jimma zone produces wheat after a government initiative. Ethiopia has a large amount of cluster‐irrigated production as a result of the government′s initiative to replace exported wheat. The low productivity and income of farmers are mainly attributed to poor adoption of irrigation [15].

Seka chekorsa district has substantial irrigation potential and provides suitable conditions for wheat production. However, farmers are not well using the available potential and did not adopt small‐scale irrigation for wheat production, as they would have expected. This could be explained by a number of factors that appear to affect farmers′ decision to adopt small‐scale irrigation wheat production [16]. Wheat production and productivity are influenced by a combination of multiple interconnected factors due to climate change, limited storage capacity, lack of grading and adjustment, existence of crop diseases and worms, price of agricultural input, lack of infrastructure, very limited access to irrigation equipment, and it depends on the traditional production system [17]. These factors contribute to the demand and supply gap [18].

Previous research, including study by Atinafu and Lejebo [19], factor influencing the adoption of improved wheat technologies and as well as works by Legesse, and Ayele [20] and Lebeta [21] on the effect of small scale irrigation on household income, did not examine how small scale‐irrigation wheat production affects household food security (measured by caloric intake). Likewise, no research has been conducted on adoption of small‐scale irrigation wheat production and it is impact on household food security in Seka chekorsa district. Therefore, we analysis the factor affecting the adoption of small scale irrigation wheat production, impact of irrigated wheat production on household food security, and the constraints and opportunities of small scale irrigated wheat production in the Seka Chekorsa district filling a research gap addressed in a previous studies.

## 2. Research Methodology

### 2.1. Description of Study Area

**Figure 1:**
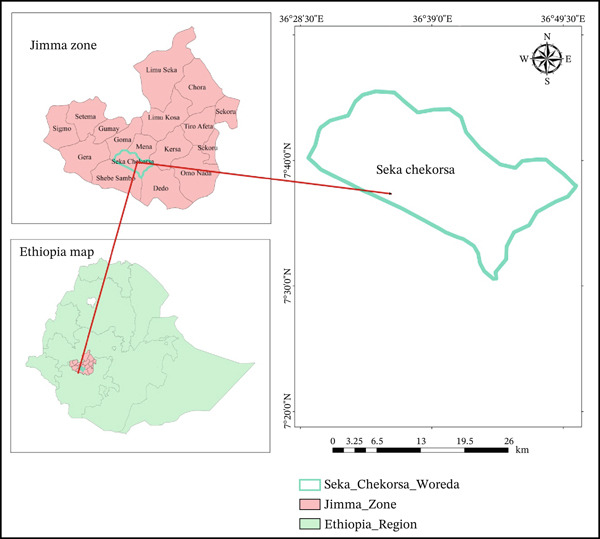
Map and location of the study area.

The study was carried out in the Seka Chekorsa district, Jimma Zone, Oromia, Ethiopia, which is around 368 km from Addis Ababa, the nation′s capital. Jimma Zone covers an area of 15,569 km^2^ and receives a mean annual rainfall of 1200–2800 mm. The district elevation varies between 1580 and 2560 meters above sea level. The latitude and longitude of this Seka Chekorsa district are 07° 35 min north latitude and 36° east longitude. The most commonly produced crops in this district are cereals such as maize, sorghum, teff, wheat, and barley. However, wheat is becoming an important crop because of its higher yield potential and commonly produced crops in the district. The main vegetable products in the area include potatoes, cabbage, green peppers, carrots, garlic, and so on. There are two agro‐ecological zones in this district. Dega (highland) and Winadega (midlands) account for approximately 18% and 82% of the total land area, respectively. The district has about 45.3% arable land of which 44.9% is annual crop, 6.1% pasture, 25.8% forest, and 22.8% wetlands. The main water source using for irrigation in the district during the dry season is the Abono, Gibe Anja, Gulufa, and Meti rivers. The common smallholder farmers in the study area used different irrigation farming systems, including motor pumps, modern micro‐dams, and traditional water diversion irrigation types used for wheat production in the district. In addition to these methods, a small‐scale irrigation scheme was constructed by a nongovernmental organization in 2022 in the Seka Chekorsa district. Figure 1below illustrated the map and location of the study area.

#### 2.1.1. Conceptual Framework

**Figure 2:**
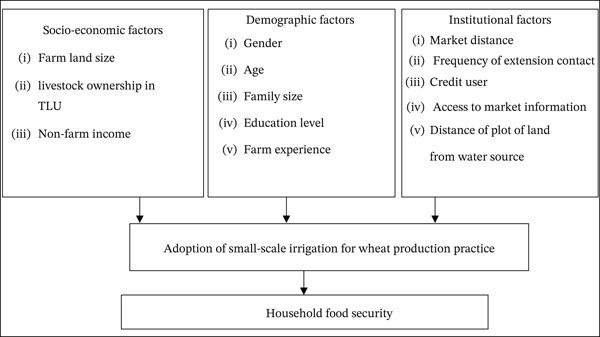
Conceptual framework of the study.

Research from different countries indicates that farmers′ adoption of small‐scale irrigation for wheat production is shaped by multiple factors. Those factors can be grouped into demographic, socioeconomic, and institutional categories that influence the adoption. A conceptual framework that takes into account household, demographic, instructional, and socioeconomic factors that may influences farmers adoption of small scale irrigation wheat production practices in the district was developed in earlier research that reviewed literature on small‐scale irrigation agricultural practices, both conceptual and empirical. A diagram of this conceptual framework is presented in Figure 2 below.

### 2.2. Study Design and Sampling Procedure

A mixed‐methods cross‐sectional survey comprising both qualitative and quantitative components was employed in this investigation. The study′s respondents and kebeles were chosen using multistage sampling techniques. Based on irrigation potential and existing practices in irrigated wheat production technology, three of the 34 kebeles were purposefully chosen in the first step after consulting with district agricultural office professionals. In the second stage, households from the three kebeles were stratified into two groups: adopters and nonadopters of small‐scale irrigation wheat production. For the third stage, 153 sample replies from nonadopters and 100 from adopters were selected at random. The families included in adopter′s strata were those who had at least 3‐year involvement status in irrigated wheat production, and the nonusers group, otherwise. Yamane′s (1967) formula was used to calculate the sample size at a 95% confidence level, 5% degree of variability, and 6% accuracy level. Due to financial and time limitation, it is not feasible to gather data from the entire population; hence, this formula with a 6% error margin was used. Finally, households were selected based on probability proportion to the size of the population in each kebele for both adopters and nonadopters.

(1) n=N1+Ne2=280712807+0.062=253

where *n* is the sample size for the study, *N* is the total number of households (2807), and *e* is the level of precision.

Quantitative data were acquired using the structured questionnaire established for this purpose. The questionnaire consists of irrigation wheat production and demographic, socioeconomic, technological, and institutional aspects of the sample households. The household food consumption and amount of food items consumed by each household in the last 7 days were also included in the questionnaire. First, the quaternaries were prepared in English and translated into the local language (Afaan oromoo) so that the enumerators could quickly and readily grasp the questions. All of them are experienced in data collection. Even though they are familiar with the study area and data collection process, half‐day training was provided on February 08, 2023, by the researchers on data collection procedures, ethics in data collection, and each section and question of the questionnaire to ensure data quality. The data were collected from February to April 2023. Moreover, to supplement the qualitative data, focus group talks and key informant interviews (KII) were used to gather qualitative data. One focus group discussion consisting of 6–8 participants was conducted in each kebele. Five KII(key informant interviews) were also conducted with development agents, model farmers, district irrigation experts, district agricultural officials, and kebele officials using an interview checklist.

### 2.3. Method of Data Analysis

The quantitative data was analyzed using both econometric models and descriptive statistics. Data analysis was done using STATA Version 15 and the Statistical Package for Social Science (SPSS) Version 20. Descriptive data was analyzed using means, standard deviations, and percentages. Additionally, *t*‐tests and chi‐square tests were employed to determine if adopters and nonadopters of small‐scale irrigation wheat production differed significantly on average. Furthermore, narrative analysis was used for qualitative data on KII (key informant interviews) and focus group discussions (FGD). The calorie conversion factors per adult equivalent were used to calculate the calorie content of food items consumed by the sample families and to 7 days of each household.

### 2.4. Econometric Model Specification

#### 2.4.1. Determinants of Small‐Scale Wheat Irrigation Adoption

The factors influencing the adoption of small‐scale irrigation wheat production and the impact of such adoption on family food security were examined using the binary logit model and propensity score matching (PSM) econometric model. A probit and a binary logistic regression model were suitable for binary variables. The decision to employ small‐scale irrigation in wheat production was considered a binary dependent variable, with a value of 1 for adopters and 0 for nonadopters. A binary logit model was chosen for the study because of its adaptability, ease of use from a mathematical perspective, and ability to produce a meaningful interpretation as explained by [22]. The following logistic distribution function was used to explain the logit model:

(2) Pi=11+ezi=Zi=βo+βixi+Ui

where *Z* *i* is between −∞ and +∞, *p* *i* is the probability of adoption of small‐scale irrigation wheat production, *β*0 is the intercepts, *β*i is the regression coefficient to be estimated, *x* *i* is the vector of household, and *U* *i* is the error term. Similarly, the probability of that household nonadopter of small‐scale irrigation wheat production is specified;

(3) 1−p=e−zi1+e−zi

Equation (3) is obtained by dividing adopters by nonadopters.

(4) Pi1−Pi=ezi 

Equation (5) is achieved by writing the natural logarithm of both sides of the equation as follows:

(5) Li=lnPi1−Pi=Zi=βo+β1122x+βx+βixi+Ui

Keep in mind that Li has linear characteristics and is the log of the odds ratio. The ratio of the likelihood that something will occur to the likelihood that it will not is known as the odds ratio. The odds ratio between the likelihood of adoption and nonadoption of small‐scale irrigation in wheat production in this scenario. Here, 1‐*P*i probability that a farmer does not adopt small‐scale irrigation in wheat production; *P*i, probability that a farmer adopts irrigated wheat production and *β*i = regression coefficient.

#### 2.4.2. Impact Estimation: PSM

To assess the impacts of adoption of small‐scale irrigation wheat production on household food security, a PSM model was used. It is a popular nonparametric techniques for assessing impacts using cross sectional data [23]. The advantage of PSM is that it does not require baseline data, the treatment assignment might not be random, and it is regarded as the second best alternative to experimental design in minimizing selection biases. PSM helps to correct for the initial difference between a cross‐section of adoption and nonadoption by matching each participant unit based on their observable features. According to Abdia, Kulasekera [24] five steps apply in PSM. These steps are predicting propensity scores, choosing a matching algorithm, identification of common support area, balancing covariate test, and sensitive analysis.

Though all five processes are crucial, the validity of PSM hinges on two important assumptions. First, the PS distribution′s common support or overlap condition suggests that comparison control observations are close to treatment observations. For that matter, we only use observations in the common support region (i.e., where the propensity score of the adopters of irrigation in wheat production is not lower than the propensity score of nonadopters and the propensity score of nonadopters is not larger than the propensity score of the adopters). The overlap in the propensity score distribution between adopters and nonadopters is compared visually. Balancing of covariates is checked using two‐sided *t*‐tests in a covariate means comparison between adopters and matched nonadopters. In addition, the propensity scores are re‐estimated on the matched sample and a likelihood ratio test for joint significance of all regressors is performed. A low pseudo *R* ^2^ in this model and a rejection of joint significance is an indication of overall balancing between adopters and matched nonadopters Caliendo and Kopeinig [25] and Lechner [26]. Second, the conditional independence (CI) assumption implies that potential outcomes are independent of using irrigation, conditional on observed covariates Lampach and Morawetz [27]. This implies that selection into using irrigation is based entirely on observable characteristics, which is a strong assumption. The robustness of our results is tested against potential violation of this assumption. For this, we follow Ichino, Mealli [28], and use a simulation‐based sensitivity analysis that simulates a binary confounder in the data to mimic a possible unobserved influential factor. Comparing these results against the baseline results allows revealing the sensitivity to violation of the CI assumption.

#### 2.4.3. Household Food Security Measurement

Measure commonly used to evaluate household food security includes undernourishment, food conception data from household surveys, dietary diversity, and estimates of food intake based on caloric acquisition methods [29]. Undernourishment serves as an indicator of national food security by comparing the average amount of available food with population needs. Household food consumption surveys collect details on family composition, such as the age and sex of members, along with information on individuals′ height and weight and activity levels [30]. Dietary data, which cover both energy intake and the quality of diets, help determine the level of food access. However, comparing results across different studies is difficult because food categories and reference periods often differ among methods [31].

This methodology effectively quantified food security by focusing on calorie intake, which is the critical measure of nutritional adequacy. By calculating the daily calories per adult equivalent, the study accounts for variations in household size and compensation, allowing for a more accurate of food access and consumption patterns. This was then compared with the recommended Kcal per adult equivalent per day (2200 Kcal) set by the Ethiopian government to determine the minimum number of calories required per adult equivalent per day to enable an adult to live a healthy and moderately active life; households that exceeded this threshold level appeared to be food secure.

### 2.5. Hypothesized Variables of Adoption of Small Scale Irrigation Wheat Production

The adoption of small‐scale irrigation wheat production is influenced by demographic, socioeconomic, and institutional factors. The variables such as educational level, farming experience, family size, credit use, extension contact, and livestock ownership are expected to positively affect adoption of irrigated wheat production, whereas age, market distance, and irrigation distance are hypothesized to have negative effects. The definitions, measurements, and expected signs of these variables are presented in Table 1.

**Table 1:** Variables selection, types, and measurement factors affecting adoption of irrigation in wheat production.

Variables	Types of variable	Measurement	Hypothesis
Gender of HH head	Dummy	1 = male, 0 = female	+/−
Age of HH head	Continues	Years	—
Farming experience	Continuous	Years	+
Education level	Continues	Formal schooling	+
Family size	Continues	Adult equivalent	+
Credit use	Dummy	1 = yes, 0 = no	+
Distance of market	Continues	Km	—
Frequency of extension contact	Continues	Number of days	+
Irrigation distance	Continuous	Minutes of walk	—
Nonfarm income	Continuous	The Ethiopian birr	+
Farm land size	Continuous	Hectare	+
Livestock ownership	Continuous	TLU	+
Access to market information	Dummy	1 = yes, 0 = no	+
Access to irrigation input	Dummy	1 = yes, 0 = no	+

*Note:* Source: authors′ computation based on empirical review.

## 3. Results and Discussions

### 3.1. Results of the Descriptive Statistics

The results of Table 2 show that the descriptive statistics of dummy variables related to household demographic and socioeconomic characteristics, institutional and technological factors. There appeared to be a significant difference based on the gender of household ahead, credit use, and access to irrigation input between both adopters and nonadopters of irrigated wheat production, whereas there was no significant difference between adopters and nonadopters of irrigated wheat production because of very high access of market information in the study area.

**Table 2:** Summary of statistics dummy variables.

Variables			Adopters (*N* = 100)	Nonadopters (*N* = 153)		Total HH (*N* = 253)		
	Frequency		%	Frequency	%	Frequency	%	(*χ* 2)
Gender	Male	94	94.00	133	86.93	227	89.72	0.07 ^∗^
	Female	6	6.00	20	13.07	26	10.28	
Credit use	Yes	58	58.00	53	34.64	111	43.87	0.00 ^∗∗∗^
	No	42	42.00	100	65.36	142	6.13	
Access to market information	Yes	94	94.00	144	94.12	238	94.07	0.97
	No	6	6.00	9	5.88	15	5.93	
Access to irrigation input	Yes	99	99.00	29	18.95	128	50.59	0.00 ^∗∗∗^
	No	1	1.00	124	81.05	125	9.41	

*Note:* Sources: own computation from survey data, 2023.

^∗^ *p* < 0.1 significant level at 10%.

^∗∗∗^ *p* < 0.01 significant level at 1%.

Table 3 displays the descriptive statistics for continuous variables associated with household, demographic socioeconomic characteristics, as well as institutional and technological factors. The results showed that age, farming experience, educational level, and family size expressed as adult equivalent, frequency of extension contact, distance of plot of land from water source, nonfarm income, farmland size, and livestock holding in TLU showed significant differences between adopter and nonadopters of irrigated wheat production, whereas no differences were found with market distance and livestock size.

**Table 3:** The summary statistics of household characteristics of continuous variables.

Variable	Total HH (*N* = 253)		Adopter (*N* = 100)		Nonadopter (*N* = 153)		
	Mean	Std. dev.	Mean	Std. dev.	Mean	Std. dev.	*t*‐test
Age of HHH (years)	38.97	8.35	40.68	9.08	37.86	7.67	−2.66 ^∗∗^
Farming experience	23.15	8.64	24.8	8.95	22.08	8.28	−2.47 ^∗∗^
Educational level	5.24	2.58	6.36	2.53	4.51	2.35	−5.94 ^∗∗∗^
Family size	4.55	1.44	5.17	1.47	4.15	1.27	−5.85 ^∗∗∗^
Market distance(km)	7.32	2.57	7.07	2.92	7.49	2.31	1.31
Frequency ext. contact	1.20	0.99	1.69	0.79	0.88	0.99	−6.85 ^∗∗∗^
Distance from the water	7.83	6.21	2.2	0.93	11.51	5.38	17.11 ^∗∗∗^
Nonfarm income	5646.84	4947.12	6627	4833.47	5006.21	4930.98	−2.58 ^∗∗^
Farm land size	1.65	0.70	1.94	0.75	1.47	0.61	−5.46 ^∗∗∗^
Livestock size (TLU)	5.80	2.06	6.07	1.68	6.24	1.82	0.75

*Note:* Sources: own computation form survey data, 2023.

^∗∗^ *p* < 0.05 significant at 5%.

^∗∗∗^ *p* < 0.01 significant at 1%.

Table 4 presents an overview of the food security status of households in the study area, comparing those who have adopted small‐scale irrigation wheat production with those who have not adopted. The results reveal that the daily average calorie intake per adult equivalent across all sample households was 2260 kcal, which implies that the average household is food secure. The daily average calorie intake per adult equivalent of adopted small‐scale irrigation wheat production was 2458.28 kcal, which was significantly higher than nonadopters (2130.57 kcal).

**Table 4:** Descriptive statistics of the household food security.

Variable	Total HH		Adopter		Nonadopter		*t*‐test
HHFS in Kcal/AE/day	Mean	SD	Mean	SD	Mean	SD	
	2260.10	600.06	2458.28	529.32	2130.57	609.72	−4.39 ^∗∗∗^

*Note:* Sources: own computation from survey data, 2023.

^∗∗∗^ *p* < 0.01 significance level at 1%.

### 3.2. Econometrics Results

#### 3.2.1. Factors Affecting the Adoption of Small‐Scale Irrigation in Wheat Production

Table 5 presents the result of the logit model estimates for factors affecting the households′ adoption of small‐scale irrigation in wheat production. Among the 13 variables hypothesized to be associated with the adoption of small‐scale irrigation in wheat production, six of them (education level, family size in adult equivalent, credit use, frequency of extension contact, distance of plot of land from water source and livestock holding in TLU) were found to be statistically significant. All of them, except distance of the plot of land from the water source positively influenced the adoption of small‐scale irrigation in wheat production.

**Table 5:** Estimation results of the logit model for the adoption of small‐scale irrigation wheat production.

Variable	Coef.	Std. Err.	Z	*p* > |*z*|	Odds ratio
Gender	1.44	0.97	1.49	0.14	4.22
Age	−0.01	0.05	−0.17	0.87	0.99
Farm experience	−0.01	0.05	−0.32	0.75	0.98
Educational level	0.34	0.11	3.09	0.00	1.40 ^∗∗∗^
Family size	0.65	0.25	2.61	0.01	1.92 ^∗^
Credit use	1.75	0.57	3.09	0.00	5.77 ^∗∗∗^
Market distance	0.03	0.11	0.30	0.77	1.03
Frequency of ext. contact	0.95	0.29	3.24	0.00	2.59 ^∗∗∗^
Irrigation distance	−0.57	0.11	−5.16	0.00	0.57 ^∗∗∗^
Nonfarm income	0.00	0.00	0.63	0.53	1.00
Farm land size	−0.89	0.55	−1.63	0.10	0.41
Livestock holding (TLU)	0.35	0.19	1.84	0.07	1.41 ^∗^
Market information	−0.81	1.29	−0.63	0.53	0.45
_cons	−5.36	2.36	−2.27	0.02	0.00
Number of obs.	253				
LR chi2 (13)	235.34	Log likelihood		−52.10	
Prob > chi2	0.00	Pseudo R2		0.69	

*Note:* Source: own computation from survey data, 2023.

^∗^ *p* < 0.1 significance level at 10%.

^∗∗∗^ *p* < 0.01 significance level at 1%.

The educational attainment of the household head was shown to have a significant impact on the livelihood of adopting small‐scale irrigation wheat production, with significant level of 1%. When other factors remain unchanged the odd of adopting this irrigation method increased by 1.40 for each additional year of schooling. This could be attributed to the fact that more educated farmers generally have better access to information and are more aware of new technologies. This increased awareness facilitates the adoption of innovation learn about the farm extension agent, social media, or various reading materials. This suggests that irrigated wheat production rises with education level as households used small‐scale irrigation. This finding conforms to [32].

Family size was to have a significant impact on adopting small‐scale irrigation wheat production with a significant level at 1%. When other factors remain constant, the odds of adopting this irrigation method increased by 1.92 as family size increased by one adult equivalent. A household with a bigger family size may afford cheaper labor due to the labor‐intensive nature of irrigated wheat production, which encourages them to switch to small‐scale irrigation wheat cultivation during the dry season. This finding confirms Wondim, Kedir [33] and Albe [34].

The use of credit was found to have a significant impact on the likelihood of adopting small‐scale irrigation wheat production, with a significant level of 1%. Credit users are 5.77 times more likely to adopt this irrigation method compared with those without access to credit. Farmers who can access credit are better equipped to obtain the necessary inputs in appropriate quantities and qualities for effective irrigation practice. This finding agrees with the work of Nakano and Magezi [35].

The frequency of extension contact significantly influenced the probability of adoption of small‐scale irrigation in wheat production at a 1% significance level. The likelihood of adopting small‐scale irrigation for wheat production increased by 2.59 with each additional contact with a development agent per month. This may be attributed to the fact that farmers who maintain a regular communication with agricultural development agents gain greater access to information regarding technology and farming advice, which subsequently affected their decision to implement small‐scale irrigation practice in wheat production. This finding agrees with Geddafa et al. [36].

The distance of a plot of land from a water source was identified as having a negative and significant impact on the likelihood of adopting small‐scale irrigation for wheat production, with a significant level at 1%. This suggests that for every additional walking minute that a plot of land is from the irrigation water source, the likelihood of adopting small‐scale irrigation for wheat production decreases by a factor of 0.57. Household with farmland situated far from the water source are less likely to implement small‐scale irrigation wheat production. This is due to the distance a plot of land is from the main irrigation source, which leads to increased financial costs and takes time to transport water to individuals′ plots for irrigation purposes. This conclusion aligns with research conducted by Mango et al., Makate [37], and Muleta and Girmay [38].

Furthermore, higher livestock holding (TLU) has significantly influenced the probability of adopting small‐scale irrigation wheat production at the 10% significance level. When other factors remain constant, the odds of adopting this small‐scale irrigation wheat production increased by 1.14 as the number of livestock increased by one tropical livestock unit (TLU). This is recognized for two reasons: First, households with a larger number of livestock have a greater chance of using the income from livestock or livestock product sales for expanding irrigated wheat production than small numbers of livestock owners. Second, rather than using contemporary machinery for plowing the field, farmers in the district rely on oxen for traction power. These findings agree with those reported by Wondimu [39].

#### 3.2.2. Impact of Adoption of Small‐Scale Irrigation in Wheat Production on Household Food Security

Table 6 summarizes the primary PSM, findings on how adopting small‐scale irrigation for wheat production influences household food security. Before estimating the average treatment effect on treated ATT, all required procedures were completed and the key assumption were verified. This included estimating propensity score, select an appropriate matching method. Identified the common support region testing balance after matching and interpreting the outcome. A sensitive analysis also conducted to determine whether the results were affected by unobserved bias. The common support region or overlap condition, for the estimate propensity scores was defined using the summary statistics of both adopters and nonadopters. As shown in Table 6, adopter propensity scores range from 0.045 to 0.997, with an average of 0.682, whereas nonadopters scores range from 0.002 to 0.901, with an average of 0.207. Therefore, the common support falls between 0.045 and 0.901.this implies that households with scores below 0.045 or above 0.901were excluded from the matching process. Following this criteria, 29 households were discarded from the impact assessment.

**Table 6:** Distribution of estimated propensity scores for sample households.

Group	Observation	Mean	STD	Min	Max
Total households	253	0.395	0.338	0.002	0.997
Adopter households	100	0.682	0.263	0.045	0.997
Nonadopter households	153	0.207	0.235	0.002	0.901

**Figure 3:**
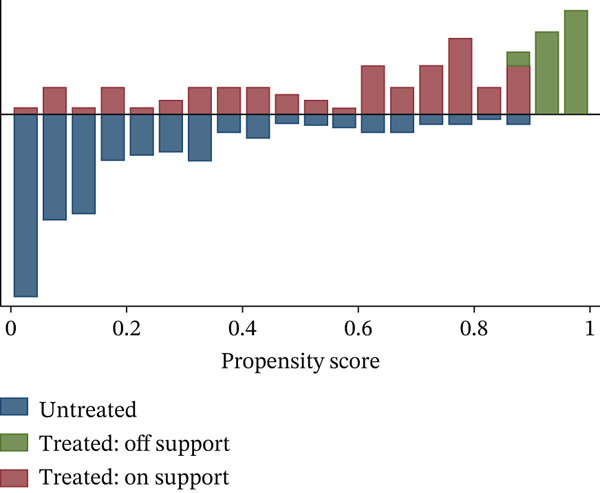
Propensity score distribution and common support region.

Figure 3 illustrates the graphical distribution of propensity scores and the identified common support region. In the histogram, the lower portion represents the propensity score distribution for nonadopter households, whereas the upper portion shows that of households adopting small‐scale irrigation in wheat production. The red bars (treated on support) and blue bars (untreated on support) indicate adopter and nonadopter observations, respectively, that have appropriate matches for comparison. In contrast, the green bars (treated off support) represent adopter observations that lack suitable counterparts within the common support region.

##### 3.2.2.1. Choice of the Matching Algorithm.

Different alternative matching estimators were tested to match the treatment and control households. To identify the most appropriate matching estimator among nearest neighbor, radius, caliper, and kernel matching, several performance criteria must be considered. This includes achieving insignificant covariate balancing test, obtaining a low *R* ^2^ value, and retaining a large matched sample size. The preferred matching methods is one that successfully balances all explanatory variables between the treated and control groups indicated by no significant mean deference while also producing the low pseudo *R* ^2^ and persevering as many observations as possible. Table 7 presents the results of the matching quality assessment evaluated using the performance criteria earlier. Finally, a kernel matching bandwidth of 0.25 was found to be an estimator for the study.

**Table 7:** Performance criteria for the selection of matching algorithms.

Performance criteria			
Matching estimator	Pseoudo‐*R* ^2^	Balancing test	Matched sample size
NN			
NN (1)	0.071	11	224
NN (2)	0.045	12	224
NN (3)	0.044	12	224
NN (4)	0.051	12	224
NN (5)	0.059	11	224
Caliper			
0.1	0.071	11	224
0.25	0.071	11	224
0.50	0.071	11	224
Radius			
0.1	0.296	7	224
0.25	0.296	7	224
0.50	0.296	7	224
Kernel			
With band width of 0.1	0.033	12	224
With band width of 0.25	**0.031**	**12**	**224**
With band width of 0.5	0.060	12	224

*Note:* Bolded values under kernel matching the with band width of 0.25 performance criteria are significant. Source: own computation from survey, 2023.

##### 3.2.2.2. Testing the Balancing Propensity of the Propensity Score and Covariates.

The balance of the propensity score and explanatory factors was examined by the chosen matching algorithm (kernel matching with bandwidth of [0.25] in this case) after the best performing matching algorithm that meets the previously established performance criteria was chosen. Matching algorithms were used to conduct balance tests of the covariates, as shown in Table 8. Additionally, it shows the covariate′s balancing test results by contrasting the significant differences before and after the matching method. Numerous factors differed significantly between the two response groups prior to matching. Nevertheless, these significant factors were conditioned to be negligible after matching, suggesting that the two groups′ means of the covariates were balanced. The standard bias difference between explanatory variables prior to matching was between 0.5% and 90.1% in absolute value, according to the data from Table 8. Nevertheless, following matching, the absolute values of the residual standardized bias differences between explanatory variables ranged from 0.9% to 18.4%, falling short of the essential threshold of 20% as suggested by Rosenbaum and Rubin [40].

**Table 8:** Propensity score and balancing propensity test.

Variable		Mean		%bias	%reduct |*b* *i* *a* *s*|	*T*‐test	
	Sample	Treated	Control			*T*	*p* > |*t*||
Pscore	Unmatched	0.67	0.21	182.2		14.53	0.001
	Matched	0.56	0.514	17.8	90.3	1.04	0.30
Age	Unmatched	40.68	37.85	33.6		2.66 ^∗∗∗^	0.001
	Matched	39	38.16	10.0	70.2	2.66	0.52
Education level	Unmatched	6.36	4.51	75.8		0.64 ^∗∗∗^	0.001
	Matched	5.96	5.81	6.0	92.1	0.36	0.71
Gender	Unmatched	0.94	0.87	24.2		1.82 ^∗^	0.07
	Matched	0.92	0.91	2.6	89.1	0.16	0.87
Adult equivalent	Unmatched	5.17	4.15	74.0		5.85 ^∗∗∗^	0.001
	Matched	4.79	4.61	12.8	82.7	0.83	0.41
Farm experience	Unmatched	24.8	22.08	31.6		2.48 ^∗∗^	0.01
	Matched	23.02	22.16	10.1	68.1	0.62	0.53
Farm land size	Unmatched	1.94	1.47	68.6		5.46 ^∗∗∗^	0.001
	Matched	1.72	1.72	−0.9	98.7	−0.06	0.9
Tropical livestock unit	Unmatched	6.74	5.19	79.9		6.27 ^∗∗∗^	0.001
	Matched	6.10	5.94	8.3	89.7	0.54	0.58
Nonfarm income	Unmatched	6627	5006.2	33.2		2.58 ^∗∗^	0.01
	Matched	6162	6728.2	−11.6	65.1	−0.65	0.51
Frequency of extension	Unmatched	1.69	0.88	90.1		6.85 ^∗∗∗^	0.001
Contact	Matched	1.55	1.50	5.0	94.4	0.29	0.77
Credit use	Unmatched	0.58	0.35	48.0		3.75 ^∗∗∗^	0.001
	Matched	0.49	0.46	7.1	85.2	0.41	0.68
Accessing market	Unmatched	0.94	0.94	−0.5		−0.04	0.96
Information	Matched	0.93	0.97	−18.4	−3602.7	−1.20	0.23
Market distance	Unmatched	7.06	7.49	−16.4		−1.31	0.19
	Matched	7.08	6.8624	8.4	48.6	0.50	0.61

*Note:* Source: own computation from survey, 2023.

^∗^ *p* < 0.1 significance level at 10%.

^∗∗^ *p* < 0.05 significance level at 5%.

^∗∗∗^ *p* < 0.01 significance level at 1%.

Moreover, low Pseudo−*R* ^2^ value and the insignificant likelihood ratio test indicated that adopter and nonadopter households had the same distribution in the covariates after matching. Table 9 shows that the pseudo *R* ^2^ value decreased as expected and dropped from 0.398 to 0.031. Therefore, these results can authentically be used to evaluate the impact of the adoption of small‐scale irrigation wheat production on household food security among groups of households with similar observed characteristics. This enables us to compare the observed outcomes for small‐scale irrigation wheat production adoption with those of a nonadoption group sharing a common support.

**Table 9:** Joint significance of variables of the balancing property002E.

Sample	Ps R2	LR chi2	*p* > *c* *h* *i*2	Mean bias
Unmatched	0.398	135.14	0.001	58.3
Matched	0.031	6.16	0.940	9.1

Source: own computation from survey data, 2023.

##### 3.2.2.3. Estimating the Average Treatment Effect on the Treated.

As indicated in Table 10, the average treatment effect of the adoption of small‐scale irrigation in wheat production using kernel matching at a bandwidth of 0.25. The mean kilocalorie intake per AE/day was 2551.29 kcal for the matched adopters, whereas it was 2032.43 kcal for nonadopters. This indicates that the adoption of small‐scale irrigation in wheat production significantly (at the 1% level of significance) increases food security by an average of 518.86 kcal/AE/day. This implies that the adoption of small‐scale irrigation for wheat production has a positive impact for improving the food security condition of the country. This could be through directly consuming wheat and/or through selling the products and buying other food that they have not had on the farm. This outcome is comparable to that of Muleta et al. [15], and Wondimagegnhu and Bogale [41]. We can infer that there is no issue with the common support and CI assumptions based on the propensity score distribution (Figure 3), the balancing property test (Table 8), and the sensitivity analysis (Table 11).

**Table 10:** Average treatment effect on treated PSM estimation results (*N* = 224).

Variable	Sample	Treated	Controls	Difference	S.E.	*T*‐stat
HHFS Kcal/AE/day	Unmatched	2458.28	2130.57	327.71	74.49	4.40
	ATT	2551.29	2032.43	518.86	109.03	4.76^a^

*Note:* Source: own computation from survey, 2023.

^a^ Indicates significance at the 1% significance level.

##### 3.2.2.4. Sensitivity Analysis.

For matching‐based studies, sensitivity analysis is a crucial premise that needs to be supported. Once the ATT of the gathered data has been determined, it is important to determine whether the obtained ATT is effective. Sensitivity analysis was carried out on the calculated outcome variables using the Rosenbaum bounding approach with regard to deviation from the CI assumption and estimated average treatment effect using the outcome variable′s kernel matching estimator in order to look for unobservable biases. To determine if the treatment effect on the treated is sensitive to hidden biases, different levels of critical values of e (gamma) were employed based on this kernel matching estimator (bwidth 0.25). Although adopter and nonadopter households have been permitted to differ in their odds of being treated up to 200% at critical Sigma 2 in terms of unobserved covariates, Table 11 results demonstrate that the inference for the impact of the adoption of small‐scale irrigation wheat production is not changing. As a result, unobserved selection bias has no effect on the impact estimations.

**Table 11:** Results of the sensitivity analysis using the Rosenbaum bounding approach.

Outcome	*e* *γ* = 1	*e* *γ* = 1.25	*e* *γ* = 1.5	*e* *γ* = 1.75	*e* *γ* = 2
HH Kcal/AE/day	0.000	0.000	0.000	0.000	0.000

### 3.3. Qualitative Data Analysis

#### 3.3.1. Results FGD

The results of FGD mainly captured farmers′ practical experiences and day‐to‐day challenges on irrigated wheat production. Farmers emphasized that the shortage of irrigation equipment, such as motor pumps and generators, is a major constraint, forcing them to rely on expensive and unreliable rental services. Participants also responded to the high cost and limited availability of inputs, including improved seeds, fertilizers, and herbicides, which reduced their ability to achieve optimal production. In addition, farmers highlighted operational challenges such as the long distance between water sources and farm plots, which increases labor and reduces efficiency, whereas bird attacks were reported as serious issues during the off‐season when alternative food sources are scarce. Additionally, the lack of harvest and postharvesting machines was also raised as a challenge.

Although the adopter irrigated wheat production, a 43‐year‐old male during FGD said that when we compare irrigated and rained wheat production, “small scale irrigation” gives higher yield than rained due to getting suitable climate condition and sun during irrigated than rained farming system.

#### 3.3.2. The Results of KII

This indicated that limited irrigation technologies are linked to high costs, weak supply system, and lack of institutional support. They also indicated that the input delivery system was inefficient and had frequently delayed distributions. From the technical perspective. Key informant interviews explained that water‐logging problems arise due to poor drainage infrastructure and inadequate water management due to limited extension service and training. Additionally, they identified climate change, specifically erratic rainfall, as an increasing risk to the production of wheat crops.

#### 3.3.3. Triangulation of Quantitative and Qualitative Findings

##### 3.3.3.1. Small‐Scale Irrigation Constraints in Wheat Production.

In addition to Table 12 presents the summary of the constraints of small‐scale irrigation wheat production in the study area. Accordingly, low availability of irrigation farming equipment was the first ranked constraint receiving a vote of 33.6%, lack of improved seed variety and high cost of agricultural input (seed, chemical fertilizers, and herbicides) was second by 19.4%, long distance between irrigation water and farm fields was third by 15.8%, irrigation water logging problems, bird attacks, premature and prolonged rains, and lack of harvest and postharvest machines were the other production constraints reported by both adopter and nonadopter respondents. This implies that small‐scale irrigation wheat production was constrained by low availability of irrigation farming equipment such as motor pumps, generators, and tradable pumps. Many smallholder farmers used the motor pump irrigation system based on rental arrangements from others. This finding agrees with the results of Ashine [42], and Mude [7]. The unavailability of improved seed, the price of agricultural inputs, like the cost of fertilizers, improved wheat seed, and the high price of motor pumps with fuels are also problems. The rising cost of inputs over time is consistent with the finding of Tadesse et al.[43]. The study also shows that irrigated wheat production lacks a reliable inputs delivery system, which causes delays and shortages in farming access to necessary inputs.

**Table 12:** The major constraints of small‐scale irrigation wheat production.

Constraints	Response	Freq	Percentage	Rank
Low availability of irrigation farming equipment′s	Yes	85	33.60	i
Lack of improved seed and high cost of agricultural input	Yes	49	19.37	ii
Long distance between irrigation water and farm fields	Yes	40	15.81	iii
Irrigation water logging problems	Yes	26	10.28	iv
Bird attack	Yes	25	9.88	v
Premature and prolonged rains	Yes	16	6.32	vi
Lack of harvest and postharvest machine	Yes	12	4.74	vii

*Note:* Source: own computation from survey, 2023.

##### 3.3.3.2. Opportunities for Wheat Production Through Small‐Scale Irrigation.

There are many advantages to irrigated wheat production for improving household food security, raising farm revenue, and lowering poverty at the individual, household, and national levels. Irrigation potential includes land and water availability, availability of the labor force, and suitability reported as opportunities. Small‐scale irrigation in wheat production in Ethiopia also creates additional job opportunities for family members, particularly for women and children, as well as for daily laborers who work on the irrigated fields of adopting households. Irrigated wheat production adopters harvest twice a year, especially those adopting subsistence food security, compared with nonadopters in the study area. The availability and suitability of river and land water are described as opportunities for irrigated wheat production. The findings indicated that the biggest possibility for expanding small‐scale irrigation in wheat production and increasing productivity is the climatic conditions with the greatest natural opportunities.

## 4. Conclusion and Policy Implications

The purpose of this study is to examine how household food security in the Seka Chekorsa district is affected by the use of small‐scale irrigation in wheat cultivation. A number of factors were found to be statistically significant, including family size, education level, credit use, frequency of extension contact, irrigation distance from farm land, and the amount of cattle kept in the TLU were found to be statistically significant. All of them, except irrigation distance, positively influenced the adoption of small‐scale irrigation in wheat production. The impact of adopting small‐scale irrigation in wheat production on household food security was examined using the PSM kernel‐matching algorithm of bandwidth 0.25. The findings indicated that adopters and nonadopters had significantly different levels of family food security. Small‐scale irrigation wheat production adopter households received an average of 518.86 kcal/AE/day compared with nonadopter households. Thus, the sample households′ estimated average treatment effect showed that adopting small‐scale irrigation in wheat production has an effect at the 1% significance level on household food security. From these results, we conclude that the households that adopt small‐scale irrigation wheat production have insured higher food security than nonadopters. The result of qualitative analysis revealed that low availability of irrigation farming equipment, lack of wheat‐improved seed, high cost of agricultural input, irrigation distance from farm fields, water logging, bird attacks, untimely rains, and lack of harvest and postharvest machine were the major constraints irrigated wheat production in the study area. However, the availability and appropriateness of river and land water are described as opportunities for irrigated wheat production. The following policy implications are forwarded for future research and development effort aimed at enhancing the adoption of small‐scale irrigation wheat production and improve household food security:

• The PSM finding shows that the adoption of irrigated wheat production had a positive and significant impact on household food security. Therefore, nonadopters should be aware of the importance of small‐scale irrigation in wheat production, and the district irrigation development office and other concerned bodies should encourage research on irrigated wheat production.
• The constraints of small‐scale irrigation wheat production in the Seka chekorsa were limited access to irrigation farming equipment, shortage of wheat‐improved seed, high cost of agricultural inputs, water logging, and bird attacks. Therefore, attention should be given to the government and its allies to continue supporting irrigated wheat production and provide agricultural input to farmers.
• The examination of the outcome variables, household food consumption score, which were measured over a seven‐day period, constituted the primary limitations of this study. To better understand the consequences of the household food consumption score in the study area, longitudinal data and additional outcome indicators should be included in future research.

## Author Contributions

Each Author participated in the study concept, design, and participated in material preparation. Zanaba Adamu Asefa led the data collection, analyzed the data, and wrote the first draft of the manuscript. Fikadu Mitiku and Yadeta Bekele Bekere supervised Zanaba Adamu, who also offered feedback on an earlier draft of the manuscript. The manuscript was reviewed by all authors.

## Funding

No funding was received for this manuscript.

## Disclosure

All authors have read and approved the final version of the manuscripts. Zanaba Adamu and Fikadu Mitiku had full access to all of the data in the study and take complete responsibility for the integrity of the data and the accuracy of the data analysis.

## Ethics Statement

The Jimma University College of Agriculture and Veterinary Medicine Research Ethical Review Board authorized the study′s instruments and methodology (Ref. RGS 2021/2023). Every single participant in the study gave their informed consent.

## Conflicts of Interest

The authors declare no conflicts of interest.

## Data Availability

The data for this study are available from the corresponding author upon reasonable request
